# Small phenolic compounds as potential endocrine disruptors interacting with estrogen receptor alpha

**DOI:** 10.3389/fendo.2024.1440654

**Published:** 2024-10-24

**Authors:** Raul Alva-Gallegos, Eduard Jirkovský, Přemysl Mladěnka, Alejandro Carazo

**Affiliations:** Department of Pharmacology and Toxicology, Faculty of Pharmacy in Hradec Králové, Charles University, Hradec Králové, Czechia

**Keywords:** endocrine disruptor, xenobiotic, catechol, estrogenicity, cytotoxicity

## Abstract

The human body is regularly exposed to simple catechols and small phenols originating from our diet or as a consequence of exposure to various industrial products. Several biological properties have been associated with these compounds such as antioxidant, anti-inflammatory, or antiplatelet activity. Less explored is their potential impact on the endocrine system, in particular through interaction with the alpha isoform of the estrogen receptor (ERα). In this study, human breast cancer cell line MCF-7/S0.5 was employed to investigate the effects on ERα of 22 closely chemically related compounds (15 catechols and 7 phenols and their methoxy derivatives), to which humans are widely exposed. ERα targets genes *ESR1* (ERα) and *TFF1*, both on mRNA and protein level, were chosen to study the effect of the tested compounds on the mentioned receptor. A total of 7 compounds seemed to impact mRNA and protein expression similarly to estradiol (E2). The direct interaction of the most active compounds with the ERα ligand binding domain (LBD) was further tested in cell-free experiments using the recombinant form of the LBD, and 4-chloropyrocatechol was shown to behave like E2 with about 1/3 of the potency of E2. Our results provide evidence that some of these compounds can be considered potential endocrine disruptors interacting with ERα.

## 1 Introduction

Estrogen receptor (ER) is a ligand-activated nuclear receptor occurring in two isoforms (α and β). An additional transmembrane estrogen receptor (G protein-coupled estrogen receptor) has been recently described (1, 2). These receptors bind the endogenous hormone estradiol (E2) but can recognize a wide variety of different compounds both of endogenous and exogenous origin (3, 4). ER ligands bind to the ER leading to receptor homodimerization. This complex is then translocated into the nucleus. The recruitment of co-activators facilitates the binding of the ER complex to the DNA. This binding happens at specific DNA sequences called estrogen response elements (ERE) (5). Estrogenic regulation can be mediated through both genomic and non-genomic pathways (6) and can occur in different time ranges (from seconds to hours) depending on the mechanism (2, 6). At a cellular level, ERα is associated with cellular growth and development. Additionally, it is involved in multiple physiological processes, such as sexual maturation, fertility, and the development of female secondary sexual characteristics (1, 7). Although E2 is the natural high-affinity ligand, many different exogenous compounds (natural or synthetic xenobiotics) have been reported to interact with the receptor (8). Such interactions can disrupt the normal homeostatic control of the endocrine system (9–11). Substances able to interact with ER and other receptors involved in the endocrine function are usually denominated endocrine disruptive chemicals (EDC) (8, 12).

**Figure 1:**
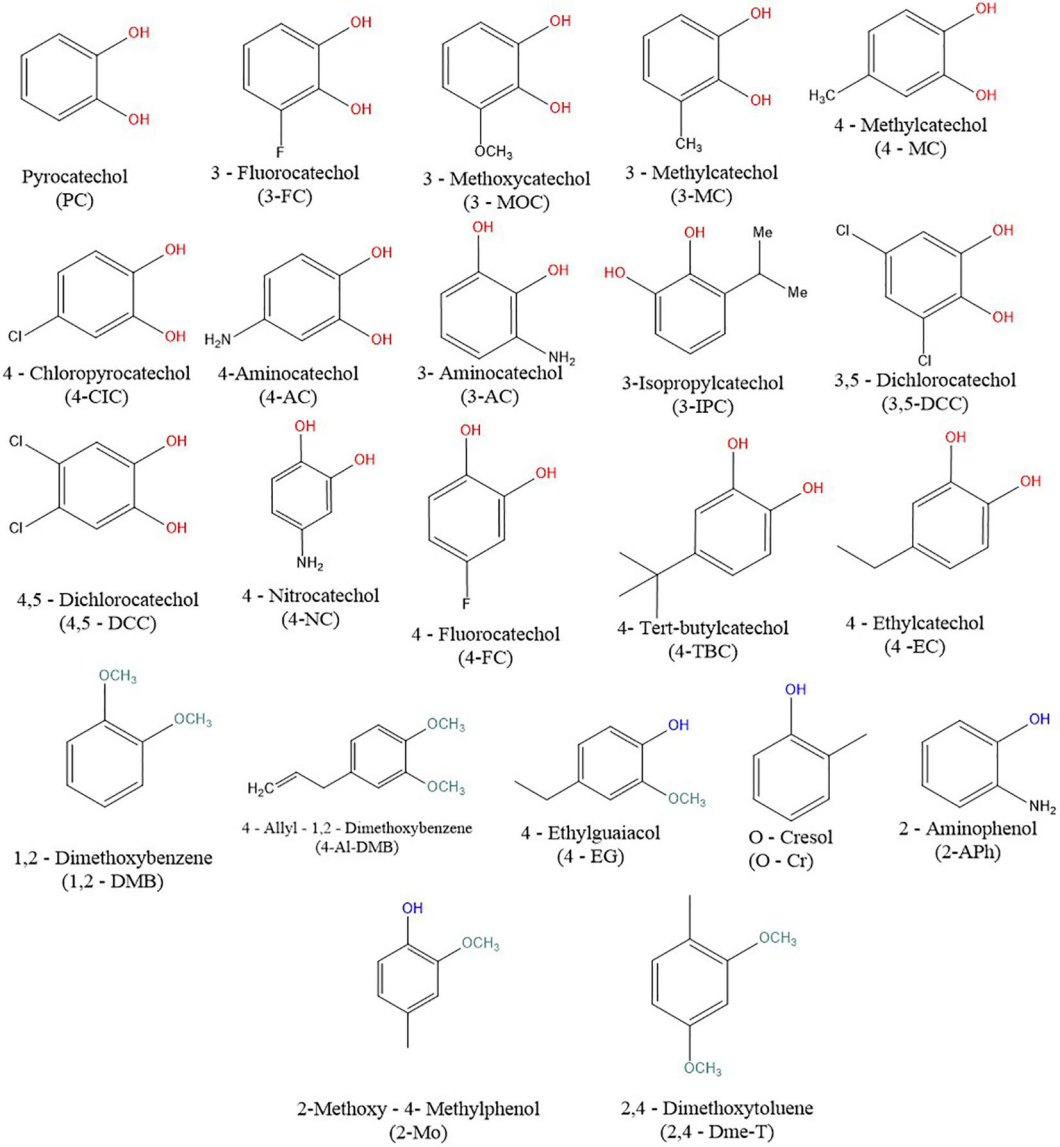
Chemical structures of the tested compounds. Red is for the hydroxyl group from the catechols, blue hydroxyl groups of the studied phenols, and green for the methoxy groups of their derivatives.

Catechols, phenols and their close benzene derivatives are exogenous substances abundantly present in wine, tea, berries, beer, and other widely used products such as cigarettes (13–15). In addition, some of these simple compounds are produced by human microbiota from other food polyphenols and they can be indeed detected in the human organism (16). Hence, the exposure of the human population to these compounds is significant. Structurally, these compounds a benzene ring. The main difference lies in the number and/or position of the hydroxyl group(s) in catechols, phenols and other benzene derivatives (**Figure 1**). Recent clinical studies have shown the involvement of these substances in the improvement of endothelial function, coronary flow velocity, reduced platelet aggregation as well as memory/cognition (17–20). The potential use of these substances as part of pharmacotherapy and their ubiquity increases the necessity of a more detailed characterization of the effects in humans including the impact on the endocrine system and hence testing of their potential toxicity.

In this paper, we aimed to elucidate if catechols, phenols, and their methoxy derivatives, including compounds with promising pharmacological potential, can interact with ERα. Human breast cancer cell line MCF-7/S0.5 was selected for this investigation as it highly expresses this receptor. A total of 22 compounds (**Figure 1**) were tested in this model both at mRNA and protein levels to evaluate their *in vitro* estrogenic potential and cytotoxicity.

## 2 Materials and methods

### 2.1 Compounds

Estradiol, 4-methylcatechol (4-MC; ≥ 95%), 3-methoxycatechol (3-MOC; 99%), 1,2-dimethoxybenzene (1,2-DMB; 99%), 2-methoxy-4-methylphenol (2-Mo; ≥98%), 2,4-dimethoxytoluene (2,4-Dme-T; 99%), 4-allyl-1,2-dimethoxybenzene (4-Al-DMB; 99%), *o*-cresol (O-Cr; 99%), pyrocatechol (PC; 99%), 4-ethylguaiacol (4-EG; 4-ethyl-2-methoxyphenol ≥98%), 3,5-dichlorocatechol (3,5-DCC; ≥ 97%), 4,5-dichlorocatechol (4,5-DCC; 97%), 2-aminophenol (2-Aph), 4-*tert.*-butylcatechol (4-TBC; ≥99,0%) and 4-nitrocatechol (4-NC; 97%) were purchased from Sigma-Aldrich (Saint-Louis, MO, USA). 3-aminocatechol (3-AC), 4-aminocatechol (4-AC), 4-ethylcatechol (4-EC), 4-fluorocatechol (4-FC), 4-chlorocatechol (4-CIC), 3-isopropylcatechol (3-IPC), 3-methylcatechol (3-MC), and 3-fluorocatechol (3-FC) were purchased from Toronto Research Chemicals (TRC Canada; Toronto, Canada).

### 2.2 Cell culture

Human breast cancer cell line MCF-7/S0.5, an MCF-7 subline adapted to a lower serum concentration, expressing ERα was purchased from the European Cell Culture Collection (ECACC, Salisbury, UK). Cells were cultured according to the indications of the manufacturer in phenol-red free Dulbecco’s Modified Eagle Medium/F-12 (Thermo Fisher Scientific, Waltham, MA, USA) supplemented with 1% FBS charcoal-stripped, 1% penicillin/streptomycin and 6 ng/mL insulin (Sigma-Aldrich) maintained at 37°C and 5% of CO_2_.

### 2.3 Cytotoxicity assays

Cell viability assays were performed using CellTiter 96^®^ AQueous One Solution Cell Proliferation Assay (Promega, Madison, WI, USA). This assay uses the bioreduction of tetrazolium salt of MTS (3-(4,5-dimethylthiazol-2-yl)-5-(3-carboxymethoxyphenyl)-2-(4-sulfophenyl)-2H-tetrazolium) into a colored formazan derivate which takes place solely in the mitochondria of living cells. Experiments were performed according to manufacturer instructions. Cells were seeded at a density of 80 x 10^3^ cells per well in a 96-well plate. After 48 hours, cells were treated with DMSO 0.1% as the vehicle control or 10% sodium dodecyl sulfate (SDS) as the positive control (causing cell death) or test compounds at low (1 µM), high (10 µM) and very high (100 µM) concentrations, and incubated for 48 h. After this period, 20 µL of MTS reagent was added to the cells and incubated for an additional 4 h. At the end of this incubation period, absorbance was measured at a wavelength of 490 nm using a microplate reader (Hidex Sense Beta Plus, Hidex Oy, Turku, Finland). Results are expressed as relative cell viability considering vehicle control samples with DMSO at 0.1% concentration as 100% viability. Experiments were performed in three technical replicates and repeated at least in three independent experiments.

### 2.4 RT-PCR

MCF-7/S0.5 cells were seeded in 48-well plates at a density of 24 x 10^4^ cells/well, and after 48 h of incubation, cells were treated with test compounds at concentrations of 10 and 50 µM for an additional 48h. RNA isolation was performed using TRI Reagent^®^ (Invitrogen, Carlsbad, CA, USA), and RNA purity was determined based on the 260/280 nm absorbance ratio. cDNAs were synthesized from 1 µg of the total RNA using a Tetro cDNA Synthesis kit (Bioline, UK) at 45°C for 30 min in the presence of oligo (dT)18 primer mix. The reaction was terminated by a heating step (85°C for 5 min). Next, cDNA was subjected to qRT-PCR performed in a QuantStudio™ 6 Flex Real-Time PCR System cycler (Applied Biosystems, Massachusetts, USA). *ESR1* (ERα) and *TFF1* mRNA expression in MCF-7/S0.5 cells were studied for that purpose using TaqMan probes (Generi Biotech, Hradec Králové, Czechia). qRT-PCR for each gene of interest was performed in at least three independent experiments performed in triplicates. Target gene expression was normalized against the reference gene β-actin and then processed using the 2^–ΔΔCt^ method. Data are presented as fold changes in the activation of gene expression relative to the vehicle-treated (DMSO 0.1%) samples set to 1.

### 2.5 Western-blot analysis

The relative expression of ERα and TFF1 specific proteins in 25– 50 μg of MCF-7/S0.5 cells total lysates was evaluated using Western blot method. MCF-7/S0.5 cells were seeded on a 24-well plate at a density of 5 x 10^5^ cells/well. After a 48 h incubation period, cells were treated with test compounds at concentrations of 10 and 50 µM. E2 10 µM and DMSO 0.1% were used as positive and vehicle (negative) controls, respectively. Incubation was terminated at 48 h, when the growth medium was removed, and cells were washed twice with ice-cold phosphate buffered saline (PBS). Cell lysis was performed using RIPA buffer (200 µL/well) and incubation on ice for 15 min. Lysates were then sonicated thrice for 15 s with 1-minute incubation on ice, centrifuged for 15 min (13 000 x g at 4°C), and supernatants were stored at -80°C until analyzed. From each sample, 10 µg of protein was incubated for 5 minutes at 95°C with 2x Laemmli buffer with 5% (v/v) β-mercaptoethanol, separated on 12% stain-free SDS-PAGE (TGX Stain-Free FastCast Acrylamide kit 12%, Bio-Rad Laboratories, Inc., California, USA) and transferred to 0.22 µm nitrocellulose membrane (Amersham™ Protran^®^, Cytiva, Marlborough, USA) using Trans-blot turbo system (2.5 A, up to 25 V, 3 min; Bio-Rad). Membranes were blocked for 1 hour using bovine serum albumin in tris-buffered saline with 0.05% Tween 20 (TBS-T; Sigma-Aldrich). Rabbit monoclonal anti-ERα antibody (ab16660, Abcam, Cambridge, UK) was used for the detection of ERα while TFF1 rabbit monoclonal antibody was used for protein pS2 detection (ab92377, Abcam), both at a dilution of 1:2000 in TBS-T for 1 hour. Goat F(ab’)2 anti-rabbit IgG (HRP) (ab6112, Abcam) antibody (dilution 1: 10 000, in TBS-T for 1 hour) was used to recognize primary antibodies using ECL Clarity substrate (Clarity Western ECL Substrate, Bio-Rad). Protein loading was normalized on total protein content using a stain-free method. Membranes were analyzed using ChemiDoc Imaging System and ImageLab software (Bio-Rad), and bands were digitized for analysis by the program Imagen Lab (Version 6.0.1 Standard edition, Bio-Rad Laboratories, Inc. California, USA). The assay was performed in two independent biological replicates. Original gels with annotation are presented in **Supplementary Data**.

### 2.6 ERα-ligand binding domain assay

LanthaScreen^®^ TR-FRET ER Alpha Coactivator Assay (Thermo Fisher Scientific) was performed according to manufacturer instructions. This assay uses two fluorophores: a terbium-labeled anti-GST antibody interacting with the human ERα ligand binding domain (LBD) and a fluorescein-labeled coactivator peptide. The interaction of an agonist results in a conformational change with an energy transfer to the acceptor fluorophore and a FRET emission shift from 495 nm to 520 nm. Thus, the energy transfer is detected by an increase in the fluorescence emission of the acceptor and a decrease in the fluorescence emission of the donor. Selected compounds were tested at concentrations ranging between 0.1 nM and 1 mM. E2 and DMSO 0.1% were used as the positive and vehicle (negative) controls, respectively. Fluorescence was measured using a Tecan Spark plate reader (Tecan Trading AG, Mannedorf, Switzerland) after a 4 h incubation period at room temperature in the dark. To quantify the process, values for DMSO 0.1% were set to 1.

### 2.7 Statistical analysis

Experimental data are presented as mean ± s.d. One-way analysis of variance (ANOVA) with the Dunnett’s multiple comparison test was performed to analyze significance. All statistical tests were performed using GraphPad Prism, version 10.1.2 (GraphPad Software, Inc., Boston, MA, USA). A p-value<0.05 was considered significant.

## 3 Results

### 3.1 Cytotoxicity

**Figure 2:**
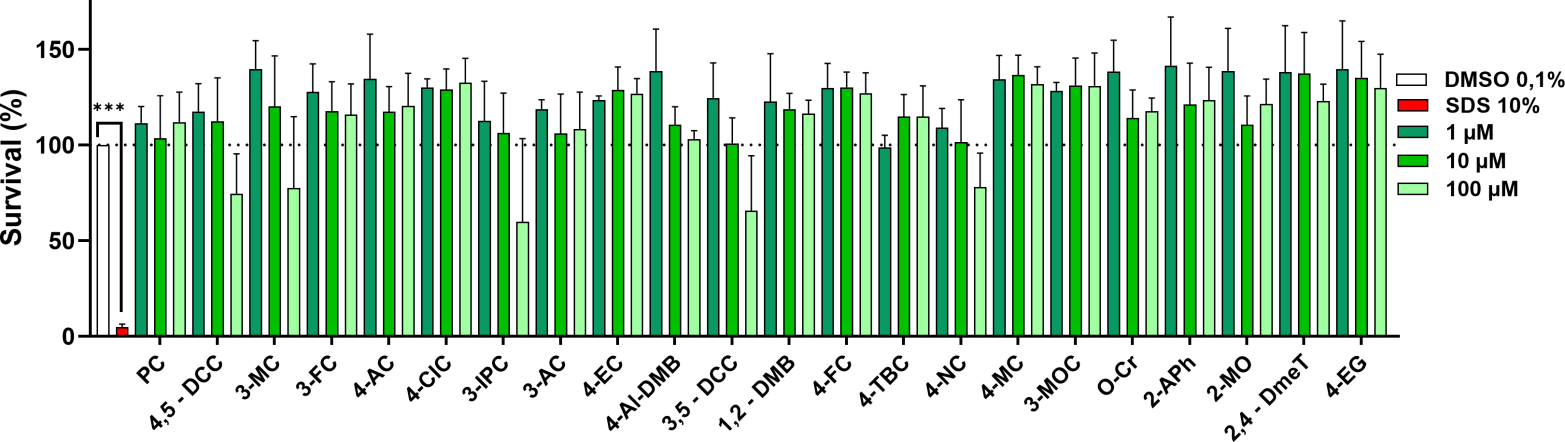
Evaluation of the viability of the selected compounds in MCF-7/S0.5 cells after a 48-h incubation at three different concentrations (high - 100 µM, medium - 10 µM, and low - 1 µM). None of the compounds significantly affected the viability. Five compounds tended to yield some degree of toxicity at the highest concentration tested. Data are presented as the mean ± s.d. of at least three independent experiments performed in triplicates. Vehicle samples (DMSO 0.1%) were set to 100% survival (dotted line) and SDS 10% was included as the positive control. One-way ANOVA statistical assay was performed with the Dunnett´s multiple comparisons *post-hoc* testing (***p<0.001).

The first step in our study was to assess the potential cytotoxicity of all 22 selected compounds on our MCF-7/S0.5 cell model. Cells were treated with concentrations of 1, 10, and 100 µM for every investigated compound, vehicle (DMSO 0.1%), or SDS 10% for a period of 48 h. Our results indicated that none of the assayed compounds was toxic to the cells at concentrations 1 and 10 µM. Most compounds were also non-toxic at a concentration of 100 µM. Only 5 of them (3-IPC, 3-MC, 3,5-DCC, 4,5-DCC, and 4-NC) tended to yield some degree of cytotoxicity (**Figure 2**). Numerically the survival rates were around 70% with the exception of 3-IPC, which yielded 60% viability. Nevertheless, these effects were not significant, similarly to a mild proliferative effect observed for nearly all tested molecules.

### 3.2 Gene expression

**Figure 3:**
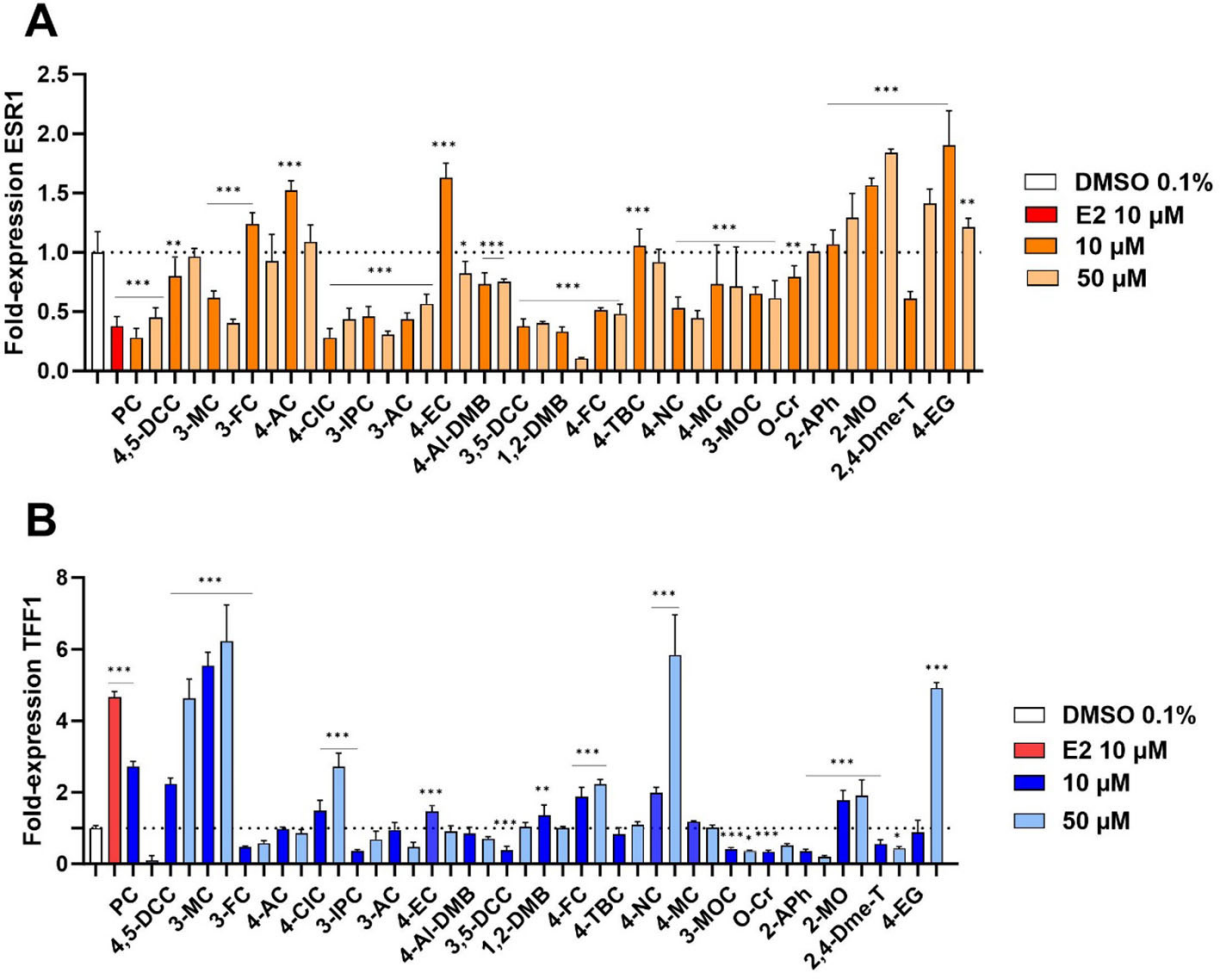
Expression of mRNA of the ER target genes *ESR1* and *TFF1* in MCF-7/S0.5 cells. A total of 6 compounds (4,5-DCC, 3-MC, 3-FC, 4-CIC, 4-FC, and 4-NC) significantly influenced the expression of these genes in a similar way as E2. qRT-PCR experiments were performed in MCF-7/S0.5 cells with TaqMan primers with DMSO 0.1%, estradiol (E2), or compounds for 48h. *ESR1* **(A)** and *TFF1* **(B)** expression was analyzed using RT-qPCR. Data are expressed as fold mRNA expression relative to vehicle-treated control cells set to 1. Expression data were normalized to β-actin reference gene values and the delta-delta method was used for data analysis. Compounds were tested at concentrations of 10 and 50 µM. Data are presented as mean ± s.d. of at least three independent experiments performed in triplicates. One-way ANOVA statistical assay was performed with the Dunnett´s multiple comparisons *post-hoc* testing (*p<0.05, **p<0.01, ***p<0.001).

ERα target genes *ESR1* and *TFF1* were selected to study the effect of the selected compounds on the receptor (21, 22). Based on our toxicity results, two concentrations were chosen to perform these experiments: 10 and 50 µM. Whereas *TFF1* codes for trefoil factor 1, *ESR1* is the gene coding for the receptor ERα itself. In the presence of E2, the expression of *ESR1* is downregulated in a process of negative feedback to avoid overstimulation. Therefore, compounds interacting with ERα are expected to show a decreased rate of mRNA expression for this particular gene. As our results showed (**Figure 3A**), most of the compounds tested decreased the expression of *ESR1* in MCF-7/S0.5 cells. This decrease was almost always more pronounced for the highest concentration tested (50 µM) than for the lower concentration (10 µM), confirming this negative feedback effect. On the other hand, *TFF1* expression was expected to follow a classical pattern, the higher the interaction of the compound with the receptor, the higher the expression observed. Results are presented in **Figure 3B**. A total of 6 compounds out of 22 tested showed an enhanced mRNA expression, some of them (3-MC, 4-NC, 4-EG), even more strongly than the endogenous ligand E2.

### 3.3 Protein expression

**Figure 4:**
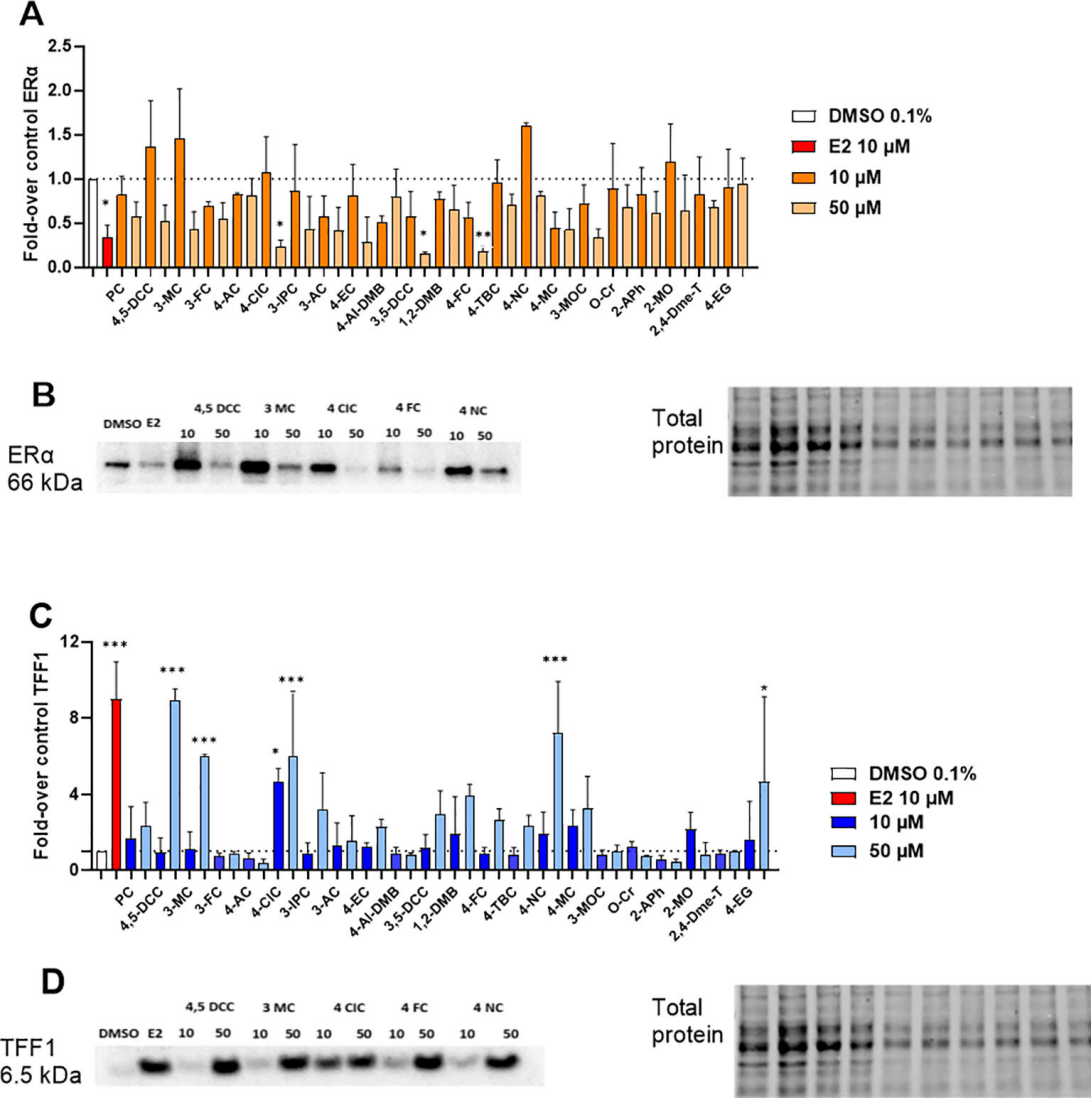
ERα and TFF1 protein expression in MCF-7/S0.5 cells. **(A)** Densitometric analysis for ERα protein expression; **(B)** Representative bands on ERα protein expression for the compounds selected for TR-FRET testing (10 and 50 µM); **(C)** Densitometric analysis for TFF1 protein expression; and **(D)** Representative bands on TFF1 protein expression for the compounds selected for TR-FRET testing (10 and 50 µM). The blank sample was DMSO 0.1%, and the positive control was E2 at a concentration of 10 μM. Results are presented as mean ± s.d. of at least two independent experiments performed in duplicates. Compounds for which western blot results are shown in this figure are underlined in red in the graphic. One-way ANOVA statistical assay was performed with the Dunnett´s multiple comparisons *post-hoc* testing (*p<0.05, **p<0.01, ***p<0.001).

In the next step, protein expression experiments were performed. Cells were treated with the compounds in the same way as in gene expression experiments, except for the number of seeded cells/well (see Materials and Methods section). Our data showed that E2 caused in general the maximal decrease in ERα protein expression. However, ERα protein expression in cells treated with other tested compounds was almost always lower than the vehicle control with the solvent DMSO 0.1%. This indicates negative feedback also in protein expression, although weaker in tested compounds when compared to the positive control E2 (**Figures 4A, B**). On the other hand, protein expression of *TFF1* gene well agreed with those obtained in PCR experiments, with a moderate protein expression. However, none of the compounds reached a protein expression as strong as E2, even at the highest concentrations tested (**Figures 4C, D**). Full membranes for protein expression of all compounds tested are presented in **Supplementary Material**.

**Figure 5:**
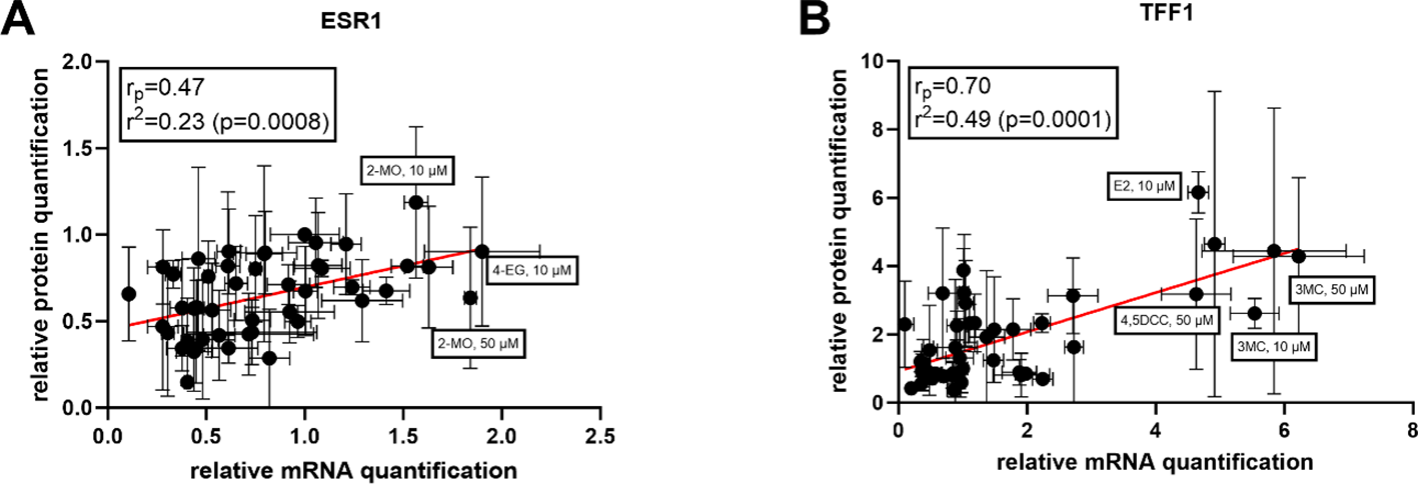
Correlations between the results of qRT-PCR and Western blot for **(A)** *ESR1* (ERα) and **(B)** TFF1.

Based on these results, we were interested to ascertain if the protein and mRNA expression results obtained for each compound could be correlated. Quite strong correlations for both genes were observed (**Figure 5**). Pearson regression values (r_p_) were 0.47 for *ESR1* and 0.70 for TFF1, indicating positive relationships between gene and protein expression, particularly for the TFF1 target gene. Overall, these results indicate that, although post-transcriptional processes occur after new protein synthesis, for most of the compounds included in this study, gene expression values can be used to predict with a good level of certainty the level of protein expression for the studied proteins.

### 3.4 ERα-ligand binding domain assay

**Figure 6:**
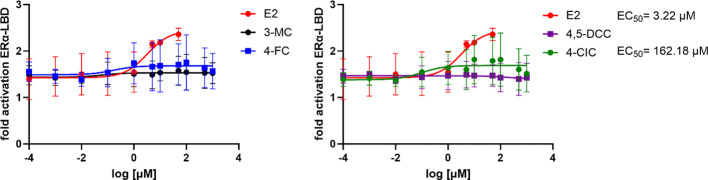
TR-FRET estrogen receptor α ligand binding domain (ERα LBD) coactivation assay with the recombinant ERα LBD for selected compounds. All results are expressed as fold activation with the vehicle-treated controls (0.1% DMSO) set to 1; the numbers are means of at least three independent experiments performed in quadruplicates ± s.d.

Based on the gene and protein expression results, we chose the 5 candidates (3-MC, 4-CIC, 4-FC, 4-NC, and 4,5-DCC) that yielded the most intense effects on genetic and protein expression to study their direct interaction with ERα LBD. For that purpose, TR-FRET assay allowing us to test the interaction of the receptors’ LBD with the test compound was employed. This assay monitors the interaction in a cell-independent manner and without the interference of additional receptor domains and factors present in cells. From the selected compounds, only 4 were finally tested since an interference with 4-NC was detected, affecting the fluorescence signal on which the test is based. All other 4 molecules were tested in concentrations ranging between 0.1 nM and 1 mM. Our results showed that, except for 4,5-DCC, the selected compounds showed to have a concentration-dependent interaction with the receptor, although relatively mild (**Figure 6**). The highest interaction was observed for 4-CIC, although the maximum reached signal was about a third of that reached by E2. These data indicate that although weak, the selected compounds can directly interact with the ERα receptor. 

## 4 Discussion

The number of xenobiotics that humans are daily exposed to is vast, particularly in highly populated and industrialized areas. They arise not only as a consequence of industrial activity but also through diet due to a growing range of chemicals used in the production, protection, and transportation of food (23). A particularly important aspect is the exposure to dietary compounds, which are gaining popularity year after year by individuals seeking to improve their health. Some of these substances of natural origin can be rich in small compounds such as simple phenolics and their derivatives. Additionally, some of these species can be formed in the organism as products of the metabolism of more complex substances (e.g. flavonoids). These compounds can be biologically active, and this activity can be positive. For instance, 4-methylcatechol (4-MC) has been proven to have marked antiaggregatory properties, being about 10 times more potent than that of the first-line antiplatelet drug acetylsalicylic acid (ASA) (17, 24). Available studies found that some small phenolic compounds including the conjugate of 4-methylcatechol with sulfuric acid are reaching units of even tens of micromolar concentrations in the human body (25). Therefore, it is feasible to assume, that the extent of biological activities of these compounds are not limited to their proven effect on platelets, and additional effects can be expected.

In light of these facts, we selected several chemically related compounds to study their potential effect as endocrine disruptors through interaction with ERα in MCF-7/S0.5 cells. We studied the potential cytotoxic activity of the selected compounds in concentrations ranging from very high (100 µM) to physiologically feasible concentrations (1-10 µM). None of the tested compounds showed important cytotoxic properties in our model, although 3-IPC, 3-MC, 3,5-DCC, 4,5-DCC, and 4-NC decreased solely insignificantly viability at a concentration of 100 µM. This is in slight disagreement with the results obtained by Fernandes et al., in which pyrocatechol showed cytotoxicity in the same cell model (26). In that study, sulforhodamine B (SRB) assay was used to establish viability, and this can be the reason for observed discrepancy. In a different study, Li et al. showed the cytotoxic effect of 4-MC in Leydig and Sertoli cells but at a concentration of 200 µM (27, 28). The difference to our results could be a consequence not only of the higher concentrations tested but also of the cell model chosen. Regardless, to avoid any potential toxic effect, non-toxic concentrations, 10 and 50 µM, were chosen for experimentation in this study.

It is known that ERα stimulation leads to decreased expression of the gene coding for this particular receptor (gene *ESR1*) thanks to a negative feedback regulatory process (29). Logically, the opposite situation occurs with other ERα target genes, like *TFF1*. Therefore, compounds decreasing ERα expression and, at the same time, enhancing that of the *TFF1* gene, can be assumed to be agonists of this particular ER receptor isoform. Our qRT-PCR results indicated that most of the tested compounds can repress ERα expression to levels lower than that of control (DMSO 0.1%) although some exceptions were observed (2-MO, 3-FC, 4-AC, and 4-EG). At first glance, a higher degree of repressive activity could be expected for the highest concentrations tested (50 µM), but this was true just for some compounds. This might indicate a potential saturation of the receptor with ligands having lower affinity to the receptor when compared to endogenous agonist E2. Indeed, our results from the ERα-LDB assay suggested a much lower affinity of the most active compounds toward ERα compared to E2 (**Figure 5**). Nevertheless, the observed results in *ESR1* mRNA and corresponding ERα protein expression did not translate into increased rates of mRNA and protein TFF1 expression since only a few compounds behaved in this way (4,5-DCC, 3-MC, 4-CIC, 4-FC, 4-NC, and 4-EG). This effect is particularly interesting for 2-MO and 4-EG. These compounds increased the expression of both studied genes in qRT-PCR assays. This phenomenon differs from that of E2 and we hypothesize it can be a consequence of a specific interaction with the receptor, which may be somehow different to that of the other compounds tested. However, future studies should focus on this particular behavior and provide a potential explanation for the observed results. In general, we have found good correlations between mRNA and protein expression indicating little or no posttranscriptional effect of most compounds. Our results hence support the theory that some of the tested compounds behave similarly to E2 and, therefore, are likely ERα agonists.

For the TR-FRET LanthaScreen^®^ assay, only the 5 most active compounds according to the previous experiments were selected: 3-MC, 4-CIC, 4,5-DCC, 4-FC, and 4-NC. The advantage of this assay is the possibility to study directly the interaction of the test compound in a cell-free manner with the LBD of the receptor, i.e. without the influence of the remaining structures of the receptor and additional proteins present in cells. Solely 4-CIC reported an ERα-LBD activation pattern similar, although weaker, to that of E2. From these data we can deduce that tested phenolic compounds can interact with other sites on the ERα or at a different level of the ERα cascade.

Even though the substances used in this project haven’t been tested for their estrogenicity before, other (poly)phenolic compounds known as phytoestrogens have shown *in vitro* to possess estrogen or even antiestrogen activity (30–32). Isoflavones such as genistein and daidzein had shown some degree of affinity towards ER (33, 34). Genistein, the most potent compound from this group, displayed estrogenicity and a higher affinity for ERβ than ERα (35, 36). In the cellular model we used, the expression of ERβ is markedly lower than that of ERα (36), therefore, the interactions here reported can be extrapolated to the ERα and not ERβ.

According to the European Food Safety Authority (EFSA) guidelines (37), and based on the results obtained in this study, 7 compounds (3-MC, 4,5 DCC, 3,5-DCC, 4,-FC, 4-NC, 4-EG and 4-CIC) from the total 22 included are candidates to be considered as potential endocrine disruptors. Nevertheless, a limitation of this study is the fact that only *in vitro* methods were employed and, therefore, additional experiments in more physiologically relevant *in vivo* models should be performed in order to confirm the findings here reported.

## 5 Conclusion

Our study found that none of the 22 tested small phenolic compounds expressed significant cytotoxicity toward breast cancer cell line MCF-7/S0.5 in concentrations ranging from 1 to 100 μM although 5 of them tended to insignificantly decrease the viability. Based on both mRNA as well as on protein expression assays, eight compounds appeared to behave in the same way as E2. Even if their effect was lower than E2, they can influence the estrogenic cascade. Direct ERα-binding assay suggested that 4-chloropyrocatechol (4-CIC) is a partial agonist at this receptor and other compounds seem to interfere with this molecular target at a different level.

## Data Availability

Raw data will be provided upon reasonable request to the corresponding author.
