# Supplementary material for: Small phenolic compounds as potential endocrine disruptors interacting with estrogen receptor alpha
Source: Front Endocrinol (Lausanne). 2024 Oct 24;15:1440654. doi: 10.3389/fendo.2024.1440654 (PMC11540614; doi:10.3389/fendo.2024.1440654)
Supplement: Supplementary file 1 [file DataSheet1.docx]

Small phenolic compounds as potential endocrine disruptors interacting with estrogen receptor alpha

Raul Alva-Gallegos, Eduard Jirkovský, Přemysl Mladěnka, Alejandro Carazo*

Department of Pharmacology and Toxicology, Faculty of Pharmacy in Hradec Králové, Charles University, Hradec Králové, Czechia

*** Correspondence:**Corresponding Author
[carazofa@faf.cuni.cz](mailto:carazofa@faf.cuni.cz)

Supplementary Material

15 pages


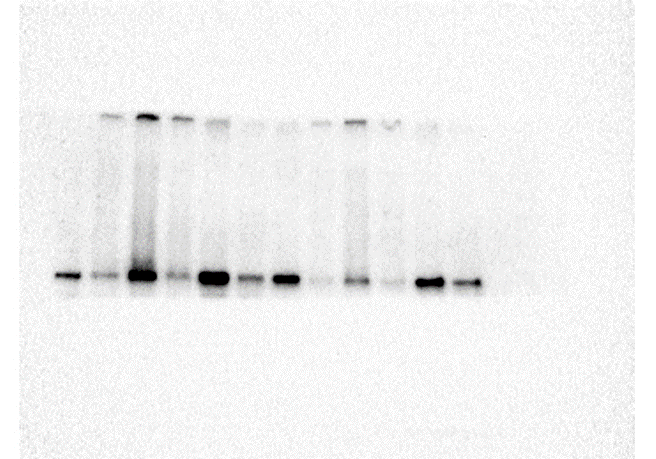


DMSO

E2

4,5-DCC

10 50

3-MC

10 50

4-CIC

10 50

4-FC

10 50

4-NC

10 50

66 kDa


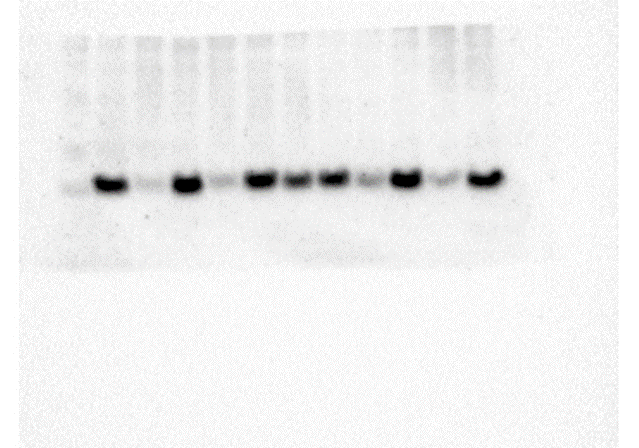


DMSO

E2

4,5-DCC

10 50

3-MC

10 50

4-CIC

10 50

4-FC

10 50

4-NC

10 50

6.5 kDa

**Supplementary Figure 1.** Membranes shown as an example for selected compounds. Top: ERα; bottom: TFF1.


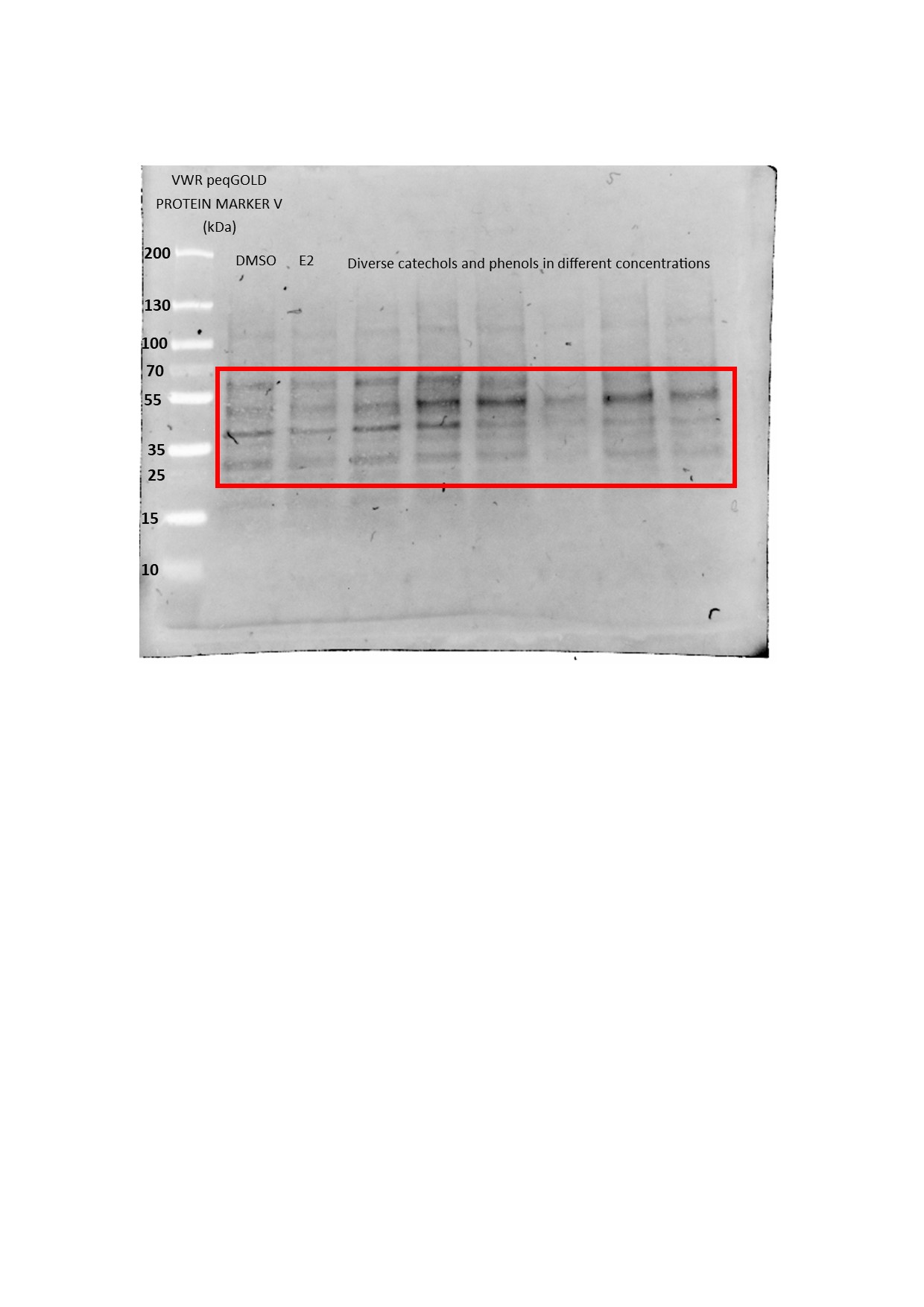


**Supplementary Figure 2.** Unedited representative result for stain-free samples. This picture represents the unedited nitrocellulose membranes used for calculation of densitometric analysis in Figure 4.


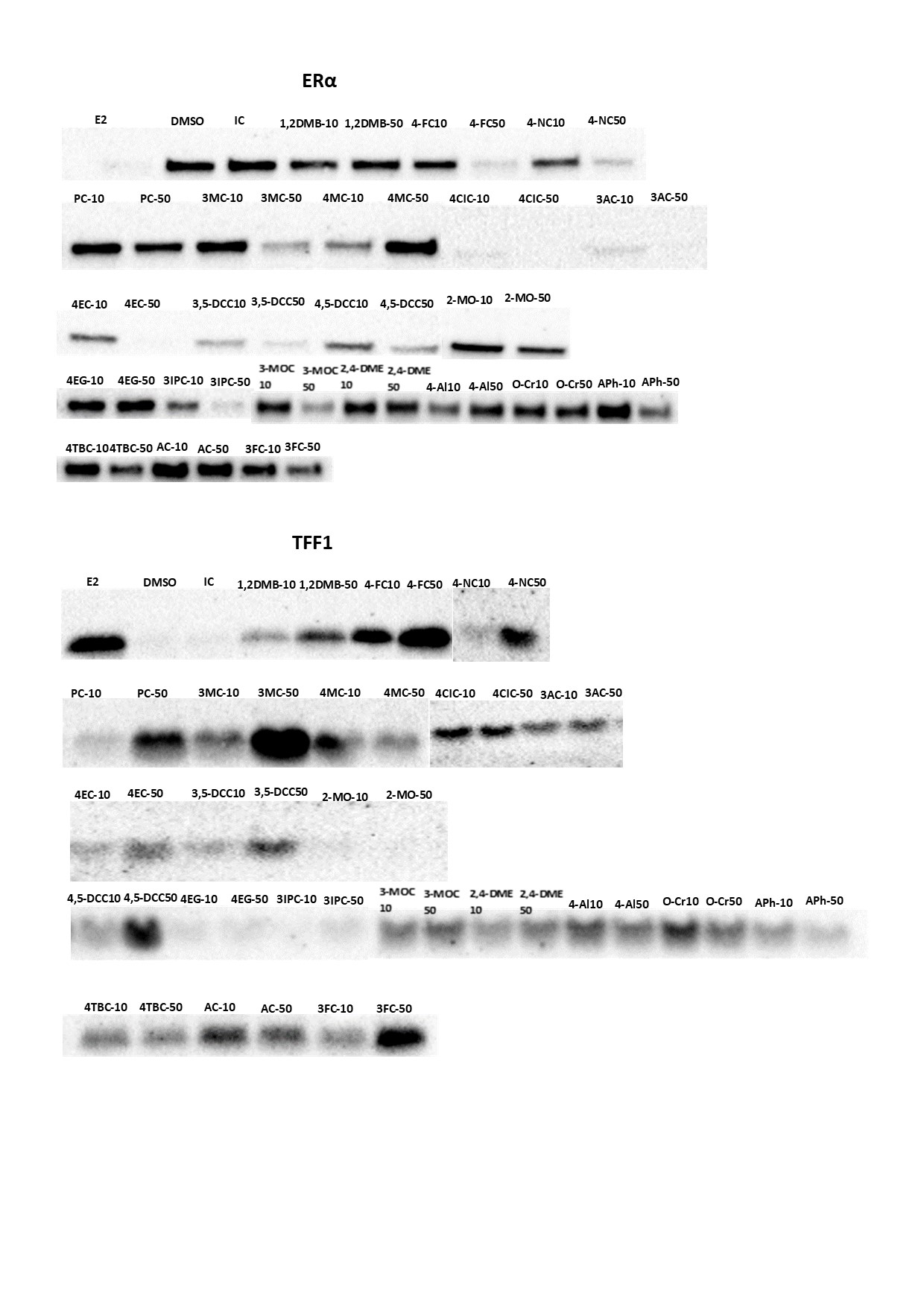


**Supplementary Figure 3.** Representative bands of all the compounds tested for both genes. These pictures are collections of all the tested compounds. Uncut images are shown below.


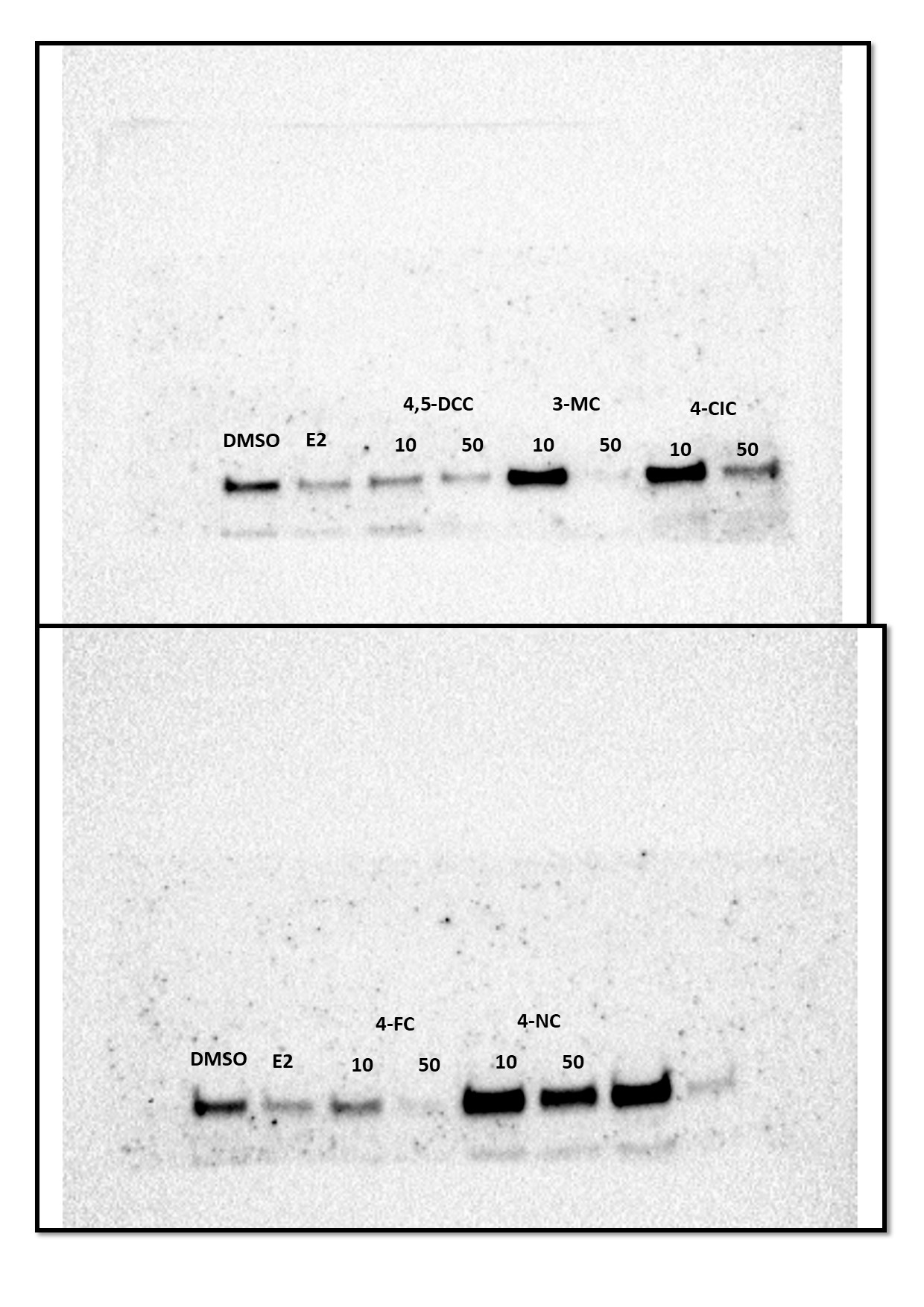


66 kDa

66 kDa

**Supplementary Figure 4.** Unedited, uncut western blot of ERα protein.

**
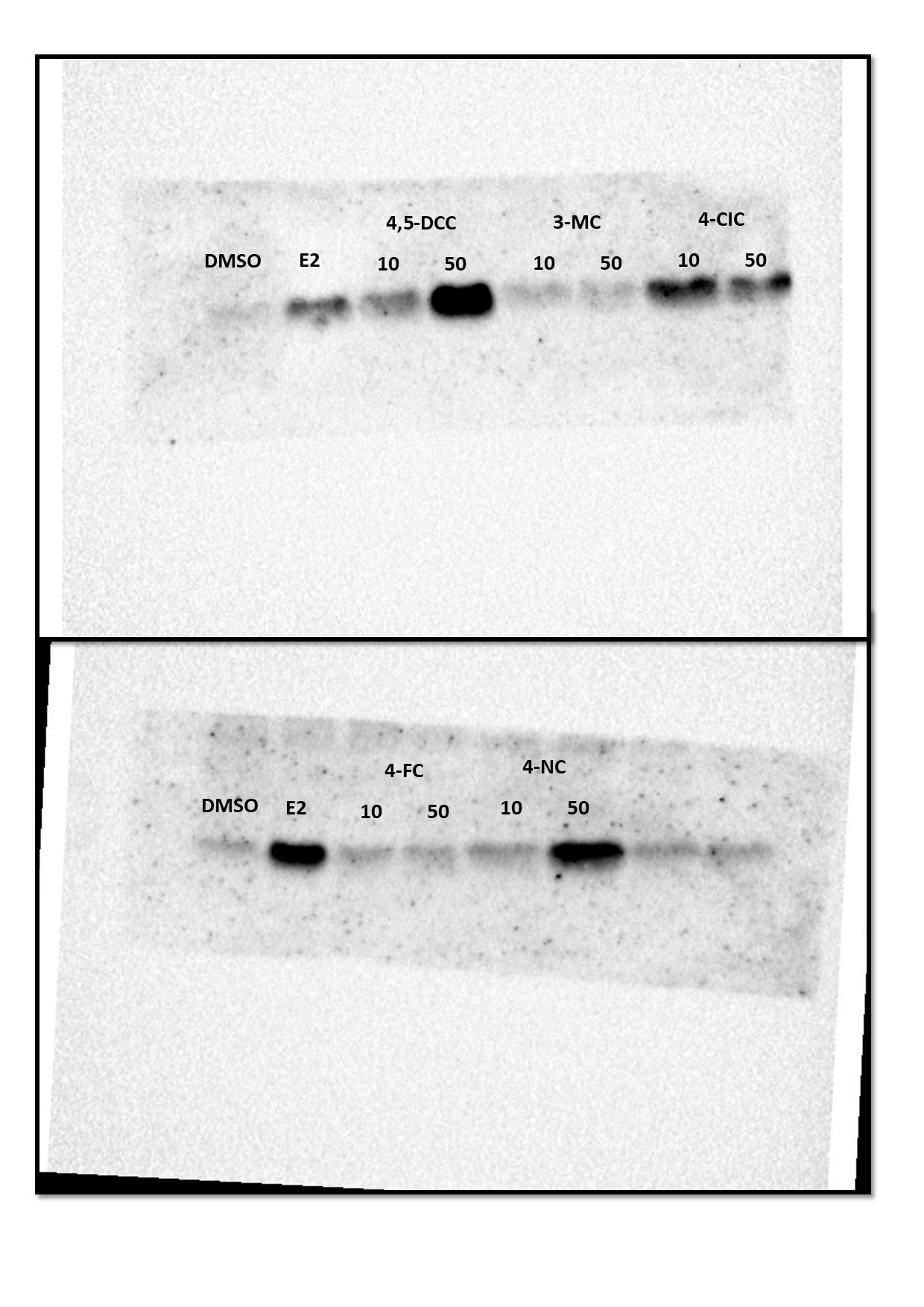
**

6.5 kDa

6.5 kDa

**Supplementary Figure 5.** Unedited, uncut western blot of TFF1 protein.


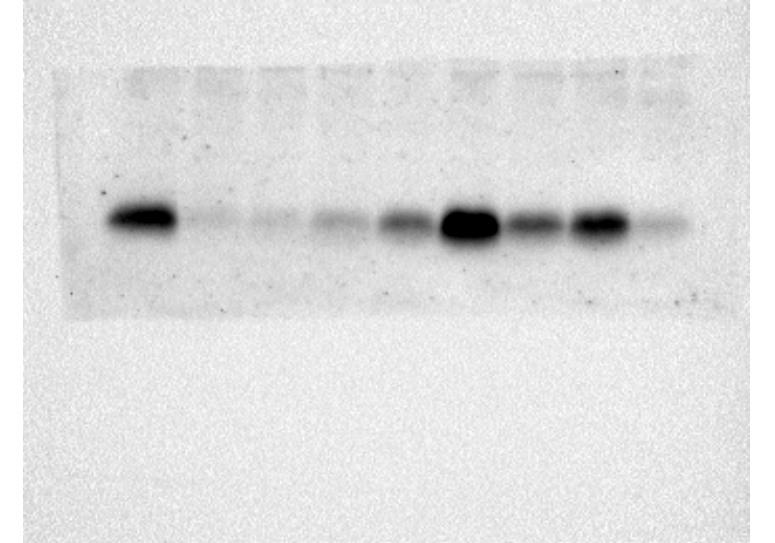

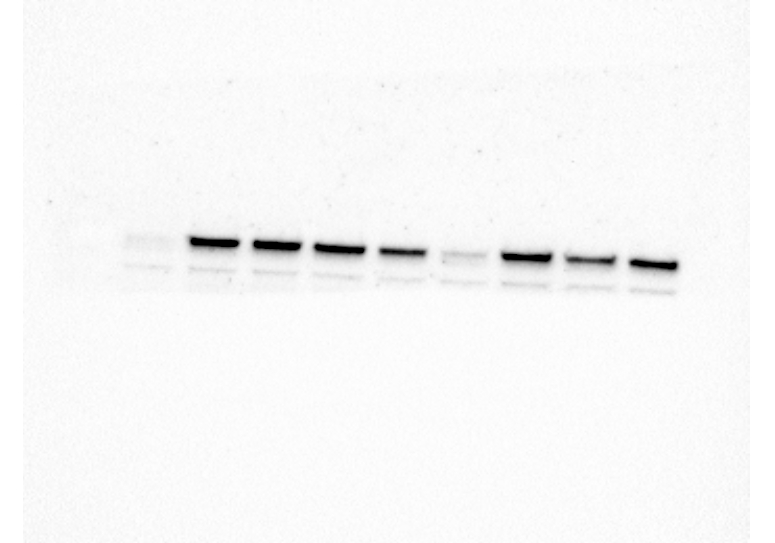

**Supplementary Figure 6.** Original pictures of representative results. Top: ERα; Bottom: TFF1

6.5 kDa

66 kDa

2MO

50

50

2MO

10

10

3MC

50

50

3MC

10

10

PC

50

50

PC

10

10

3MC

10

10

3MC

50

50

2MO

10

10

2MO

50

50

IC

DMSO

E2

PC

50

50

PC

10

10

IC

DMSO

E2


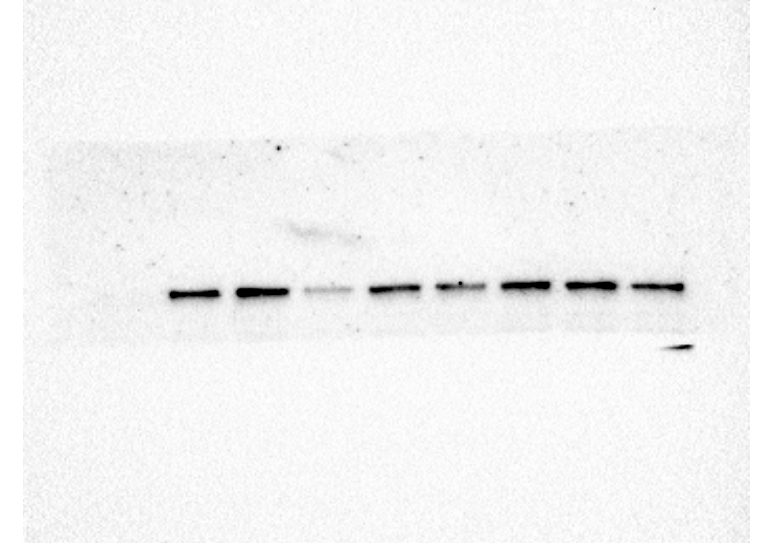


4NC

50

4NC

10

4,5DCC

50

4,5DCC

10

4EG

50

4EG

10

E2

IC

DMSO

66 kDa


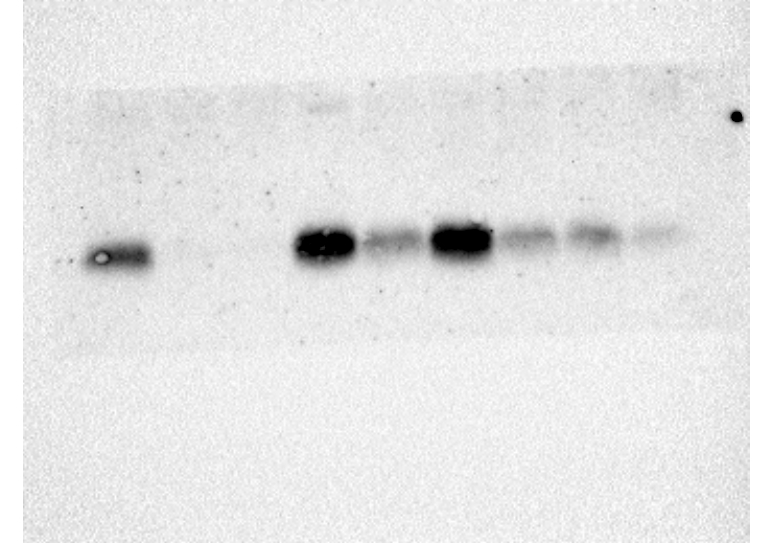


4,5DCC

50

4NC

50

IC

DMSO

E2

4NC

10

4,5DCC

10

4EG

50

4EG

10

6.5 kDa

**Supplementary Figure 7.** Original pictures of representative results. Top: ERα; Bottom: TFF1


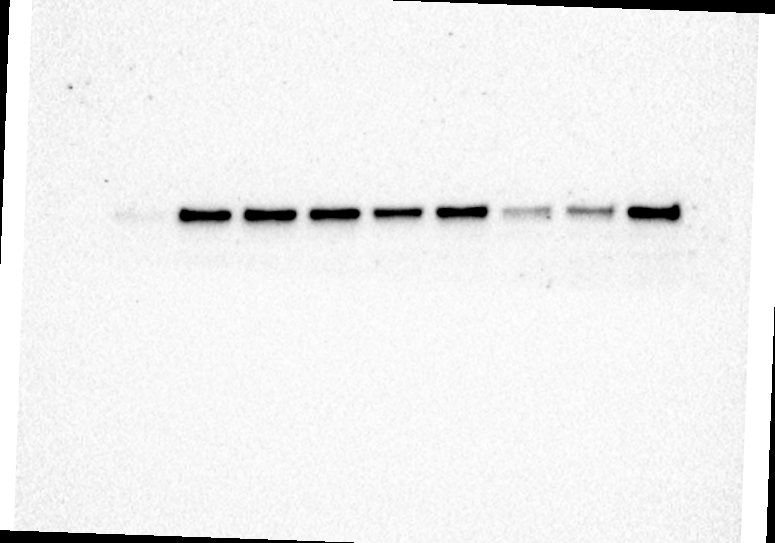


3MC

50

DMSO

IC

PC

10

PC

50

3MC

10

4MC

10

4MC

50

E2

66 kDa


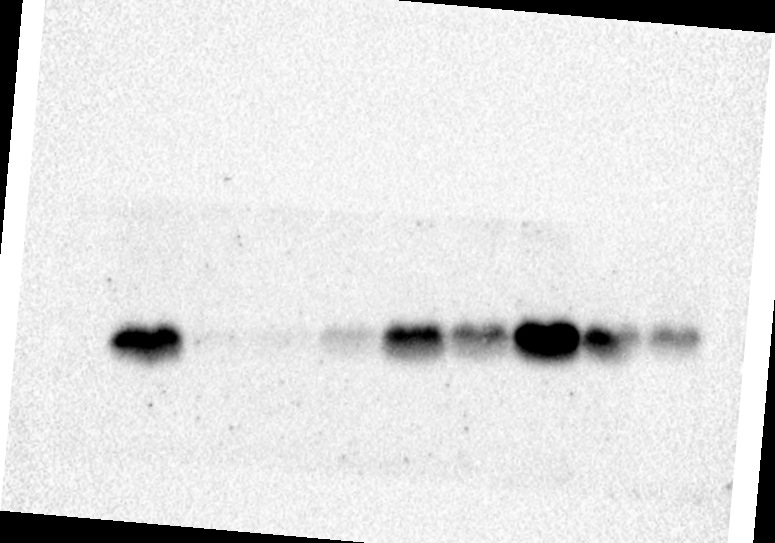


E2

DMSO

IC

PC

50

3MC

10

3MC

50

4MC

10

4MC

50

PC

10

6.5 kDa

**Supplementary Figure 8.** Original pictures of representative results. Top: ERα; Bottom: TFF1


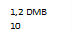

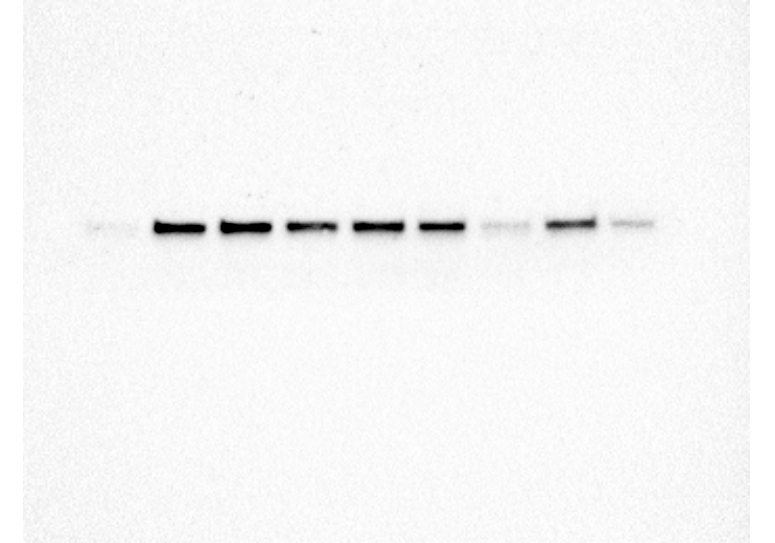


4NC

50

4NC

10

4FC

50

4FC

10

1,2 DMB

50

IC

DMSO

E2

66 kDa


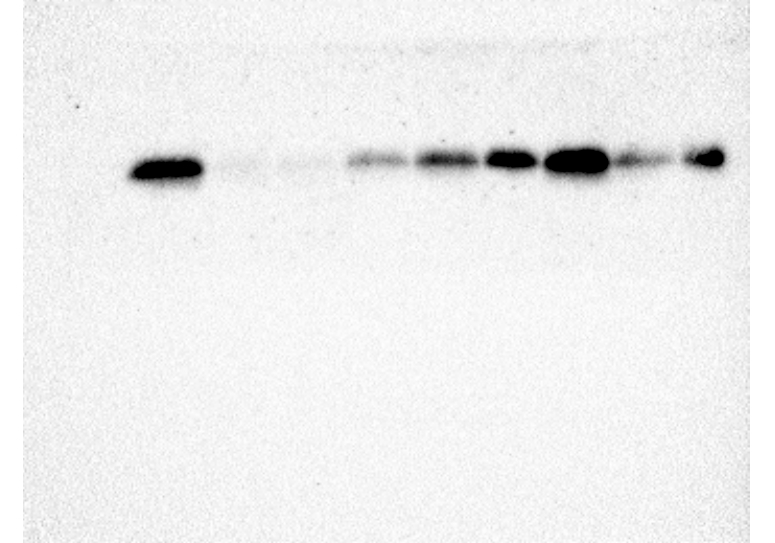


E2

4FC

10

4FC

50

4NC

10

4NC

50

1,2 DMB

50

IC

DMSO

1,2 DMB

10

6.5 kDa

**Supplementary Figure 9.** Original pictures of representative results. Top: ERα; Bottom: TFF1


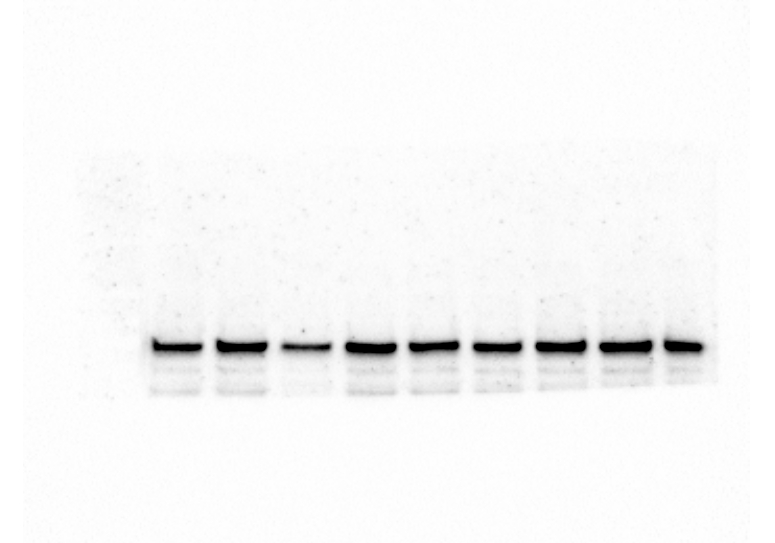


4Al

50

4Al

10

2,4DME

50

2,3DME

10

3MOC

50

3MOC

10

IC

DMSO

E2

66 kDa


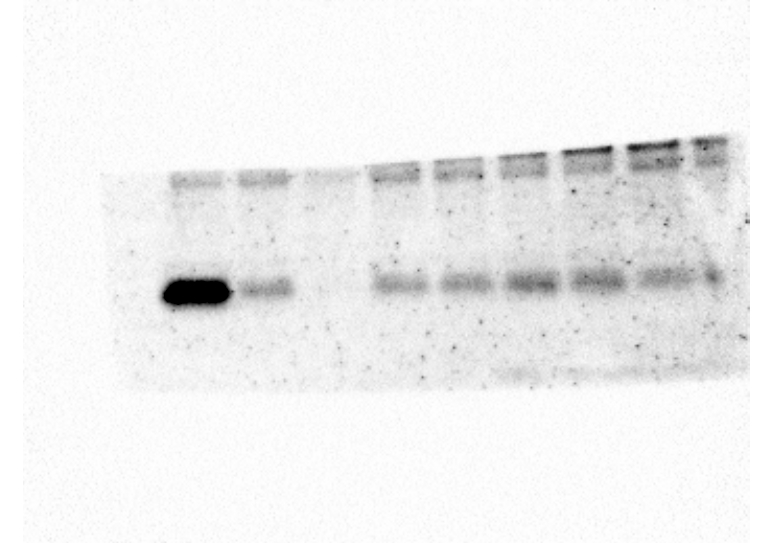


4Al

50

4Al

10

2,4DME

50

2,3DME

10

3MOC

50

3MOC

10

IC

DMSO

E2

6.5 kDa

**Supplementary Figure 10.** Original pictures of representative results. Top: ERα; Bottom: TFF1


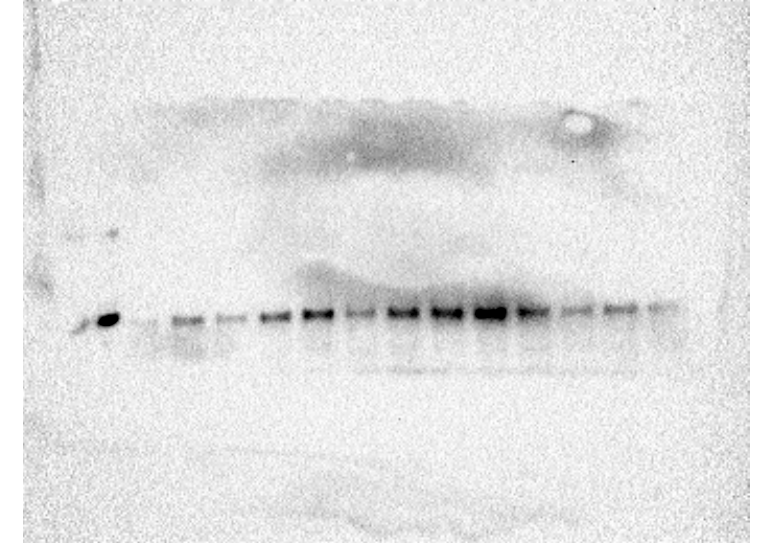


2,4DME

50

2APh

50

2APh

10

OCr

50

OCr

10

4Al

50

4Al

10

2,3DME

10

3MOC

50

3MOC

10

66 kDa

IC

DMSO

E2


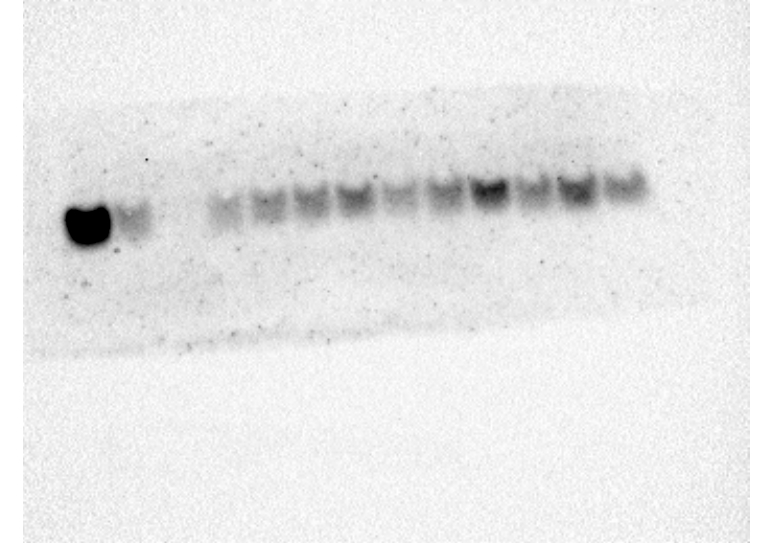


3MOC

10

2,3DME

10

3MOC

50

2,4DME

50

4Al

10

4Al

50

OCr

10

OCr

50

2APh

10

2APh

50

E2

DMSO

IC

6.5 kDa

**Supplementary Figure 11.** Original pictures of representative results. Top: ERα; Bottom: TFF1


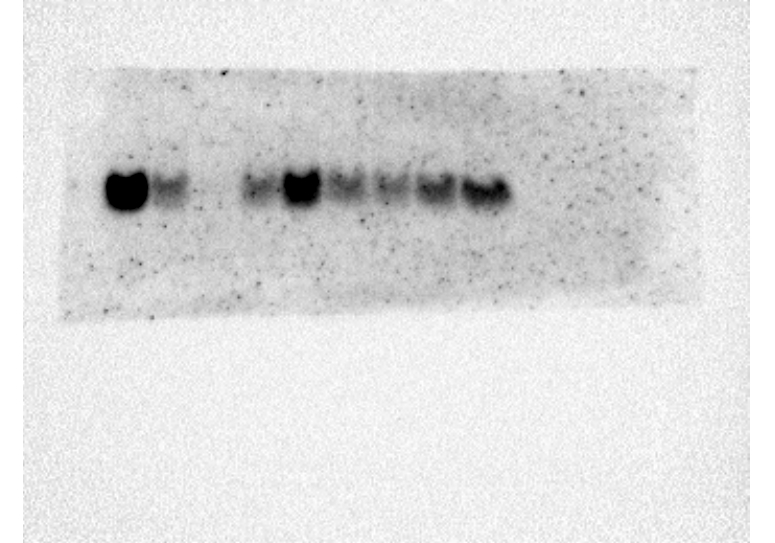

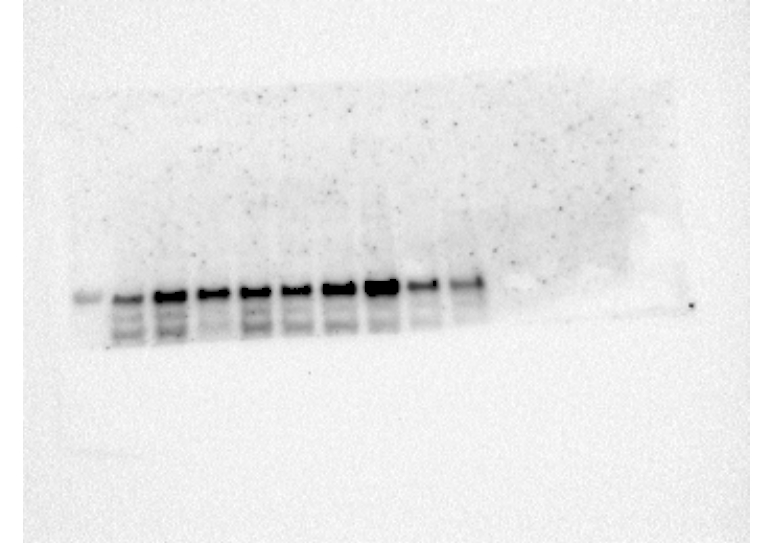


3FC

50

3FC

10

4AC

50

4AC

10

4TBC

50

4TBC

10

IC

DMSO

E2

3FC

50

3FC

10

4AC

50

4AC

10

4TBC

50

4TBC

10

IC

DMSO

E2

66 kDa

6.5 kDa

**Supplementary Figure 12.** Original pictures of representative results. Top: ERα; Bottom: TFF1


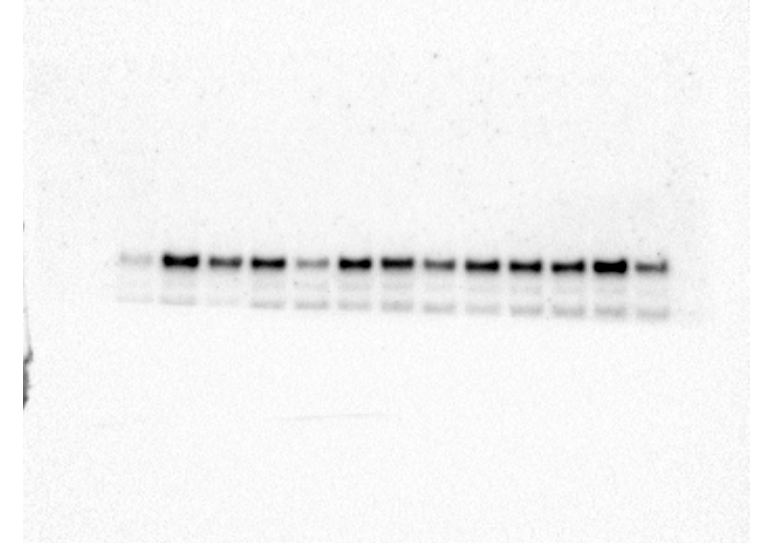


2APh

50

2APh

10

OCr

50

OCr

10

4Al

50

4Al

10

2,4DME

50

2,3DME

10

3MOC

50

3MOC

10

IC

DMSO

E2

66 kDa


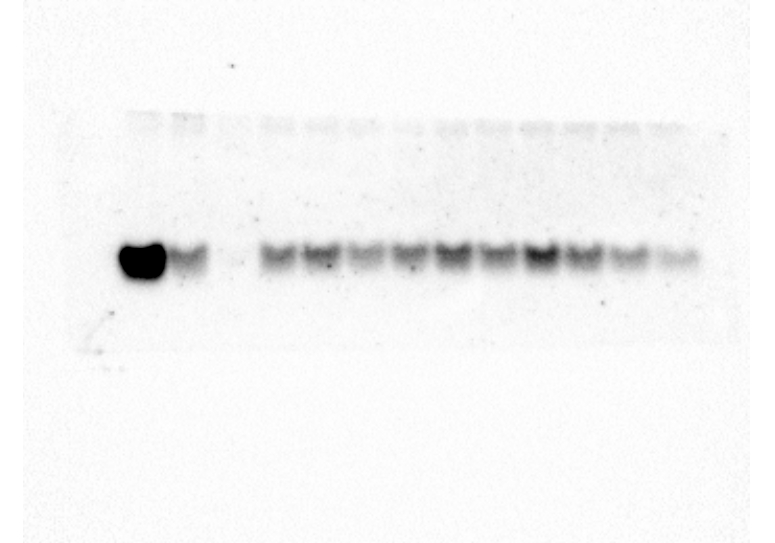


OCr

10

4Al

10

4Al

50

2,4DME

50

2,3DME

10

3MOC

50

3MOC

10

E2

IC

DMSO

2APh

50

2APh

10

OCr

50

6.5 kDa

**Supplementary Figure 13.** Original pictures of representative results. Top: ERα; Bottom: TFF1


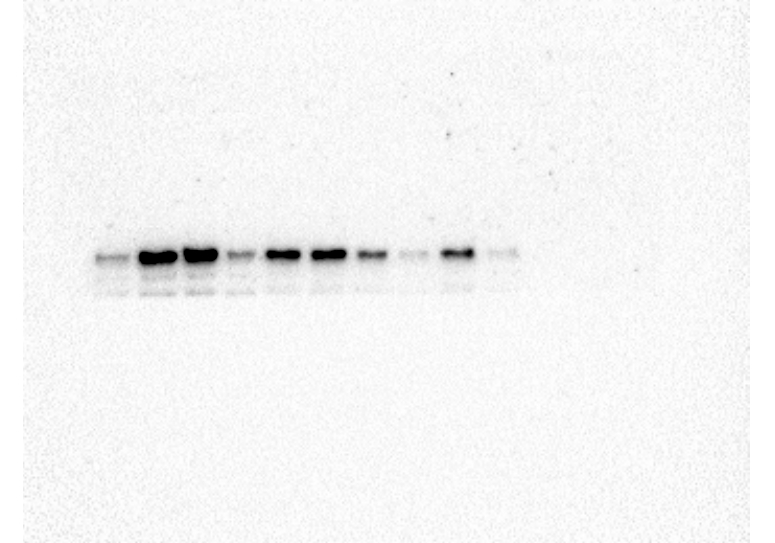


4,5DCC

10

3IPC

50

3IPC

10

4EG

50

4EG

10

4,5DCC

50

IC

DMSO

E2

66 kDa


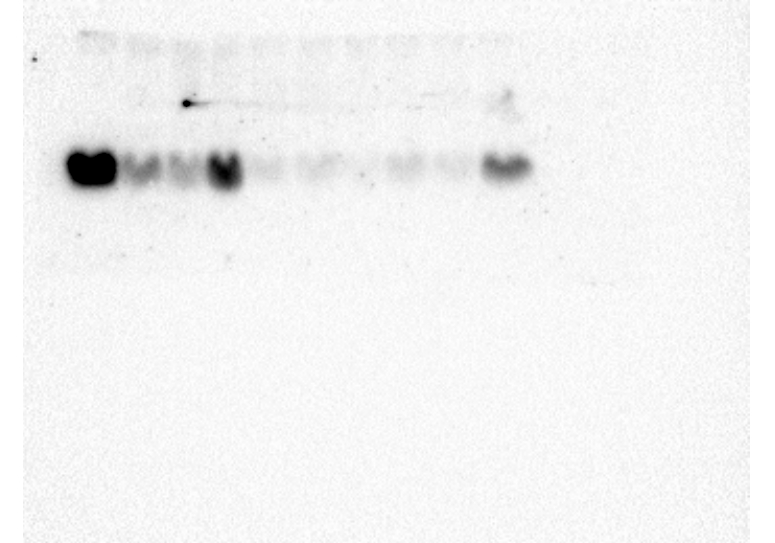


4EG

10

4EG

50

3IPC

10

3IPC

50

4,5DCC

50

4,5DCC

10

IC

E2

DMSO

6.5 kDa

**Supplementary Figure 14.** Original pictures of representative results. Top: ERα; Bottom: TFF1
